# Supplementary material for: Herd Immunity to SARS-CoV-2 Among the Armenian Population in the Second Half of 2022
Source: Epidemiologia (Basel). 2025 Jun 20;6(3):29. doi: 10.3390/epidemiologia6030029 (PMC12468988; doi:10.3390/epidemiologia6030029)
Supplement: Supplementary file 1 [file epidemiologia-06-00029-s001.zip › epidemiologia-3627021-supplementary materials.pdf]

Supplementary Materials

Table 1S. Quantitative distribution of plasma anti-Nc antibodies by age group.

Age group, years	N, persons	Anti-Nc antibody level, BAU/ml																	
		<17			17-31			32-124			125-332			333-666			>666		
		n	%	CI	n	%	CI	n	%	CI	n	%	CI	n	%	CI	n	%	CI
1–17	258	30	11.6	8.0-16.1	31	12	8.3-16.6	76	29.7	24.2-35.3	77	29.7	24.2-35.7	27	10.4	7.0-14.8	17	6.6	3.9-10.3
18–29	276	29	10.5	7.1-14.7	32	11.6	8.0-15.9	94	34.3	28.7-39.8	57	20.6	16.0-25.8	42	15.2	11.2-19.9	22	7.9	5.0-11.8
30–39	456	67	14.7	11.6-18.3	54	11.8	9.0-15.2	165	36.2	31.8-40.8	109	23.9	20.1-28.1	39	8.6	6.2-11.5	22	4.8	3.1-7.2
40–49	512	52	10.1	7.7-13.1	55	10.7	8.2-13.7	170	33.3	29.3-37.4	128	25	21.3-28.9	60	11.7	9.0-14.8	47	9.2	6.8-12.0
50–59	556	39	7	5.0-9.4	49	8.8	6.6-11.5	180	32.5	28.6-36.4	134	24.1	20.7-27.8	78	14	11.2-17.2	76	13.6	10.9-16.8
60–69	516	41	8	5.8-10.6	37	7.2	5.1-9.8	160	31	27.0-35.2	154	29.8	25.9-34.0	73	14.1	11.3-17.5	51	9.9	7.4-12.8
70^+	400	39	9.7	7.0-13.1	35	8.7	6.2-11.9	115	28.9	24.5-33.4	101	25.2	21.0-29.7	55	13.7	10.5-17.5	55	13.7	10.5-17.5
Total	2974	297	10	8.1-11.7	293	9.8	7.9-11.0	960	32.3	30.6-34.0	760	25.5	24.0-27.1	374	12.6	11.4-13.8	290	9.8	7.7-10.9

Notes: N – number of volunteers in each age group; n – number with the specified antibody level; CI – 95% confidential interval; 70^+ – 70 years and older

Table 2S. Quantitative distribution of plasma anti-RBD antibodies by age group.

Age group, years	N, persons	Anti-RBD antibody level, BAU/ml											
		<22.6			22.6-220			220.1-450			>450		
		n	%	CI	n	%	CI	n	%	CI	n	%	CI
1–17	258	8	3.1	1.3-6.0	78	30.1	24.6-36.1	66	25.5	20.3-31.2	106	41.4	35.3-47.2
18–29	276	6	2.2	0.8-4.6	81	29.2	24.0-35.0	68	24.6	19.6-30.1	121	44.0	38.1-49.7
30–39	456	13	2.8	1.5-4.8	125	27.4	23.4-31.8	105	23	19.2-27.2	213	46.7	42.1-51.4
40–49	512	11	2.1	1.1-3.8	100	19.5	16.2-23.2	108	21.1	17.6-24.8	293	57.3	52.9-61.4
50–59	556	9	1.6	0.7-3.1	79	14.2	11.4-17.4	107	19.2	16.0-22.7	361	65.0	60.9-68.8
60–69	516	11	2.1	1.1-3.8	104	20.2	16.8-23.9	84	16.3	13.2-19.8	317	61.4	57.1-65.6
70^+	400	12	3	1.6-5.2	60	15	11.6-18.8	69	17.2	13.6-21.3	259	64.8	59.9-69.5
Total	2974	70	2.4	1.8-3.0	627	21.1	19.6-22.6	607	20.4	19.0-21.9	1670	56.2	54.4-58.0

Notes: N – number of volunteers in each age group; n – number with the specified antibody level; CI – 95% confidential interval; 70^+ – 70 years and older
